# Structural insights into non-covalent ubiquitin activation of the cIAP1-UbcH5B∼ubiquitin complex

**DOI:** 10.1074/jbc.RA118.006045

**Published:** 2018-12-06

**Authors:** Amrita Patel, Gary J. Sibbet, Danny T. Huang

**Affiliations:** From the Cancer Research UK Beatson Institute, Garscube Estate, Switchback Road, Glasgow G61 1BD, Scotland, United Kingdom and the Institute of Cancer Sciences, University of Glasgow, Glasgow G61 1BD, Scotland, United Kingdom

**Keywords:** ubiquitin, ubiquitin ligase, allosteric regulation, structural biology, ubiquitin-conjugating enzyme (E2 enzyme), ubiquitylation (ubiquitination), E3 ubiquitin ligase, activation, cIAP1, non-covalent, RING E3, UbcH5B, activation, protein degradation, apoptosis

## Abstract

Ubiquitin (Ub)-conjugating enzymes and Ub ligases control protein degradation and regulate many cellular processes in eukaryotes. Cellular inhibitor of apoptosis protein-1 (cIAP1) plays a central role in apoptosis and tumor necrosis factor signaling. It harbors a C-terminal RING domain that homodimerizes to recruit E2∼Ub (where ∼ denotes a thioester bond) complex to catalyze Ub transfer. Noncovalent Ub binding to the backside of the E2 Ub-conjugating enzyme UbcH5 has previously been shown to enhance RING domain activity, but the molecular basis for this enhancement is unclear. To investigate how dimeric cIAP1 RING activates E2∼Ub for Ub transfer and what role noncovalently bound Ub has in Ub transfer, here we determined the crystal structure of the cIAP1 RING dimer bound to both UbcH5B covalently linked to Ub (UbcH5B–Ub) and a noncovalent Ub to 1.7 Å resolution. The structure along with biochemical analyses revealed that the cIAP1 RING domain interacts with UbcH5B–Ub and thereby promotes the formation of a closed UbcH5B–Ub conformation that primes the thioester bond for Ub transfer. We observed that the noncovalent Ub binds to the backside of UbcH5B and abuts UbcH5B's α1β1-loop, which, in turn, stabilizes the closed UbcH5B–Ub conformation. Our results disclose the mechanism by which cIAP1 RING dimer activates UbcH5B∼Ub and indicate that noncovalent Ub binding further stabilizes the cIAP1-UbcH5B∼Ub complex in the active conformation to stimulate Ub transfer.

## Introduction

Post-translational modification of proteins by ubiquitin (Ub),^2^ achieved via the sequential actions of Ub-activating enzyme (E1), Ub-conjugating enzyme (E2), and Ub-ligase (E3), governs vast arrays of eukaryotic cellular processes (1, 2). E1 activates and transfers the C terminus of Ub to the E2's catalytic cysteine to produce an E2∼Ub thioester intermediate (where ∼ denotes a thioester bond). E3 binds E2∼Ub and substrate to promote Ub transfer from E2 to a nucleophile, which is usually a lysine side chain. There are three major types of E3s: RING, HECT, and RING-in-between-RING (RBR) (3, 4). RING E3s harbor a RING domain that binds and activates E2∼Ub to promote the direct transfer of Ub from E2 to the substrate. In contrast, HECT E3s contain a catalytic cysteine and catalyze a two-step Ub transfer reaction in which Ub is initially transferred from E2 to HECT E3's catalytic cysteine and then to the substrate. RBR E3s share common features from both RING and HECT E3s, where a RING-like domain (RING1) recruits E2∼Ub and transfers Ub to the catalytic cysteine on RING2 prior to transfer to substrate.

Cellular inhibitor of apoptosis protein-1 (cIAP1) is a RING-type E3 and belongs to the inhibitor of apoptosis (IAP) family of proteins. The RING-mediated ubiquitin ligase activity of cIAP1 is essential for its function in both cell death and survival pathways. In cell death pathways, cIAP1 inhibits apoptosis by sequestering and ubiquitinating second mitochondria-derived activator of caspase (SMAC) for degradation by the proteasome, thereby freeing XIAP to bind and inhibit caspases (5–7). Moreover, cIAP1 has been shown to target caspases for ubiquitination and degradation by the proteasome (8). In the cell survival pathway, tumor necrosis factor receptor 1 signaling complex recruits RIP kinase 1 (RIPK1) and various adaptor proteins, including TRADD, TRAF2, and TRAF5, that lead to the recruitment of cIAP1 and cIAP2 (9). cIAP1 and cIAP2 ubiquitinate RIPK1 and components within this complex to enable the recruitment of a linear Ub chain assembly complex that ultimately activates NF-κB signaling (10–16).

cIAP1 contains three N-terminal baculoviral IAP repeat domains (BIR1–3), followed by a Ub-associated domain (UBA), a caspase-recruiting domain (CARD), and a C-terminal RING domain. Dimerization of its C-terminal RING domain is important for E2∼Ub recruitment and ligase activity (17, 18). Studies showed that the N-terminal BIR3-UBA-CARD domain sequesters the RING domain in an inactive conformation to prevent RING dimerization (19, 20). The addition of SMAC or SMAC mimetic induces conformational changes that restore activity by allowing RING dimerization (19, 21). Currently, how RING dimerization activates cIAP1's ligase activity, and the structure of cIAP1 RING domain bound to E2∼Ub, are not known. However, there are several structures of RING E3s bound to E2 covalently linked to Ub (E2–Ub; en dash denotes covalent linkage) (22–32). Collectively, these structures show that the RING domain binds and stabilizes E2–Ub in a closed conformation such that the thioester bond is optimized for Ub transfer (33). For dimeric RING E3s, such as BIRC7, an IAP family protein, the C-terminal tails of each subunit of the RING dimer function to stabilize the closed E2–Ub conformation to enhance ligase activity (23). It seems likely that cIAP1 RING dimer utilizes a similar mechanism for activating E2–Ub.

cIAP1 has been shown to function with the UbcH5 family of E2s to catalyze substrate ubiquitination (34, 35). This family of E2s has a noncovalent Ub binding site on its backside. This backside Ub-UbcH5 interaction is important for processivity of poly-Ub chain formation (25, 36–39). Our recent structural study on the monomeric RING E3 RNF38 showed that backside-bound Ub (Ub^B^) stimulates RNF38-catalyzed Ub transfer by restricting the flexibility of UbcH5B's α1 and α1β1-loop to stabilize the closed active RNF38 RING-UbcH5B–Ub complex, thereby enhancing the rate of catalysis (25). It remains unclear whether this mechanism is conserved for dimeric RING E3s.

To better understand how dimeric cIAP1 RING domain (cIAP1R) activates E2∼Ub for Ub transfer and how Ub^B^ could influence this process, we present a crystal structure of cIAP1R bound to UbcH5B–Ub and Ub^B^. Structural and biochemical analyses showed that cIAP1R forms multiple contacts with UbcH5B–Ub to stabilize it in a closed conformation. Notably, the C-terminal tail of cIAP1R functions in *trans* to stabilize the closed UbcH5B–Ub conformation, thereby explaining the importance of RING domain dimerization, and consistent with prior examples of dimeric RING E3s. Last, Ub^B^ restrains UbcH5B's α1β1-loop conformation to stabilize contacts with donor Ub (*i.e.* Ub conjugated to UbcH5B; hereafter Ub^D^). This interaction augments stabilization of the closed UbcH5B–Ub conformation, thereby enhancing Ub transfer. Our results reveal a conserved Ub^B^-stimulatory mechanism for both monomeric and dimeric RING E3s in mediating UbcH5B∼Ub transfer.

## Results

### Ub^B^ stimulates cIAP1R-mediated Ub transfer

**Figure 1.**
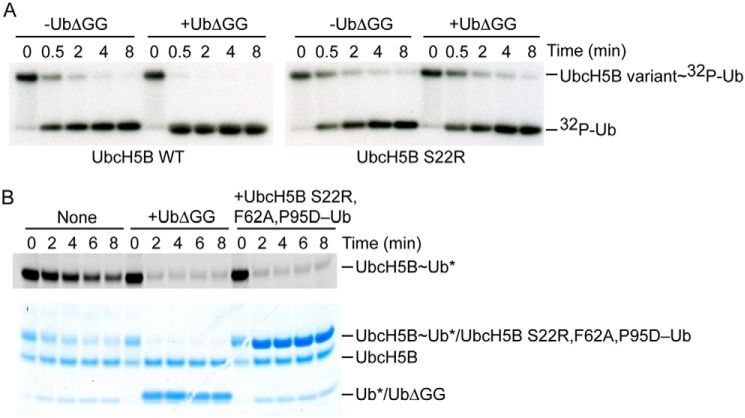
**Ub^B^ stimulates cIAP1R-catalyzed Ub transfer.** *A*, nonreduced autoradiograms of lysine discharge reactions showing the disappearance of UbcH5B variant∼^32^P-Ub over time in the presence and absence of UbΔGG (300 μm) catalyzed by cIAP1R. *B*, nonreduced SDS-PAGE showing the cIAP1R-mediated discharge of fluorescently labeled UbcH5B∼Ub to l-lysine over time in the presence of UbΔGG (20 μm) or UbcH5B S22R,F62A,P95D–Ub (20 μm) visualized with a LI-COR Odyssey scanner (*top*) followed by staining with InstantBlue (*bottom*). *, fluorescently labeled Ub.

Previously, we showed that the addition of UbΔGG (lacking the C-terminal diglycine motif) can serve as Ub^B^ and bind to UbcH5B's backside to stimulate UbcH5B∼Ub discharge catalyzed by the monomeric RING E3 RNF38 and dimeric RING E3 XIAP. To assess whether Ub^B^ can exert similar effects on cIAP1R-catalyzed Ub transfer, we performed single-turnover lysine discharge assays using WT and S22R UbcH5B. S22R substitution abrogates the Ub^B^-UbcH5B interaction and was therefore used as a control (25, 36). UbcH5B variants were precharged with equimolar concentrations of ^32^P-Ub and then chased by the addition of cIAP1R alone and in the presence of UbΔGG, which cannot be charged by E1 but can still bind to the backside of UbcH5B WT. The addition of 300 μm UbΔGG stimulated the discharge of UbcH5B∼Ub but had no effect on UbcH5B S22R∼Ub (Fig. 1*A*), indicating that Ub^B^ stimulates cIAP1R-catalyzed Ub transfer.

### Synergistic binding enhancement between Ub^B^, cIAP1R, and UbcH5B–Ub

Our prior study showed that Ub^B^ stimulates RNF38 and XIAP-catalyzed Ub transfer by enhancing RING E3 affinity for UbcH5B–Ub by ∼5–10-fold (25). To determine whether Ub^B^ functions in a similar manner to stimulate cIAP1R-catalyzed Ub transfer, we performed surface plasmon resonance (SPR) experiments to investigate the effects of Ub^B^ on cIAP1R's affinity for UbcH5B–Ub. We generated stable UbcH5B–Ub complex by mutating UbcH5B's catalytic cysteine (Cys^85^) to lysine, thereby forming a stable amide linkage that mimics the thioester linkage (22). UbcH5B C85K and UbcH5B S22R C85K covalently linked to Ub (hereafter referred to as UbcH5B–Ub and UbcH5B_S22R_–Ub, respectively) were generated to assess the effect of backside binding. cIAP1R exhibited weak binding affinity for UbcH5B alone, but displayed ∼270-fold higher binding affinity for UbcH5B–Ub (Table 1 and Fig. S1), suggesting that Ub^D^ contributes to RING domain binding, consistent with previous observations with other RING E3s (23, 25). The addition of excess UbΔGG (0.6 mm; *K_d_* for Ub^B^-UbcH5B is ∼280 μm (25)) further enhanced cIAP1R's affinity for UbcH5B–Ub by ∼4-fold (Table 1 and Fig. S1). In contrast, the addition of excess UbΔGG had no effect on cIAP1R's affinity for UbcH5B_S22R_–Ub, suggesting that Ub^B^-UbcH5B interaction enhances cIAP1R's affinity for UbcH5B–Ub.

**Table 1:** ***K_d_* values for interactions between cIAP1R, UbcH5B, UbcH5B–Ub variants, and Ub**

Immobilized protein	Analyte	*K_d_*
		μ*m*
GST-cIAP1R	UbcH5B	223 ± 4
GST-cIAP1R	UbcH5B–Ub	0.83 ± 0.05
GST-cIAP1R	UbcH5B–Ub + 0.6 mm UbΔGG	0.22 ± 0.01
GST-cIAP1R	UbcH5B_S22R_–Ub	0.90 ± 0.01
GST-cIAP1R	UbcH5B_S22R_–Ub + 0.6 mm UbΔGG	0.99 ± 0.05
GST-Ub	UbcH5B–Ub + excess cIAP1R	13 ± 2

We showed previously that Ub^B^ binds UbcH5B–Ub with a *K_d_* of ∼280 μm, but, in the presence of the monomeric RING E3 RNF38, the *K_d_* improved by 20-fold (*K_d_* of 14 μm), revealing a synergistic effect in RNF38-UbcH5B–Ub and Ub^B^-UbcH5B binding (25). Similar to our prior observation, we found that Ub displayed a *K_d_* of 13 μm for UbcH5B–Ub in the presence of cIAP1R (Table 1 and Fig. S1), suggesting that this binding synergy is conserved.

To verify the improved Ub^B^-UbcH5B interaction in the presence of cIAP1R, we performed single-turnover lysine discharge assays using 20 μm UbΔGG, which is just above the *K_d_* of 13 μm, and showed that it was sufficient to stimulate cIAP1R-catalyzed Ub transfer (Fig. 1*B*). Furthermore, we showed that UbcH5B S22R,F62A,P95D–Ub, a stable isopeptide conjugate that cannot bind RING E3 or Ub^B^ but can serve as the Ub^B^ source (25), also stimulated cIAP1R-catalyzed Ub transfer at 20 μm (Fig. 1*B*).

### Overall structure of cIAP1R-UbcH5B–Ub-Ub^B^ complex

**Figure 2.**
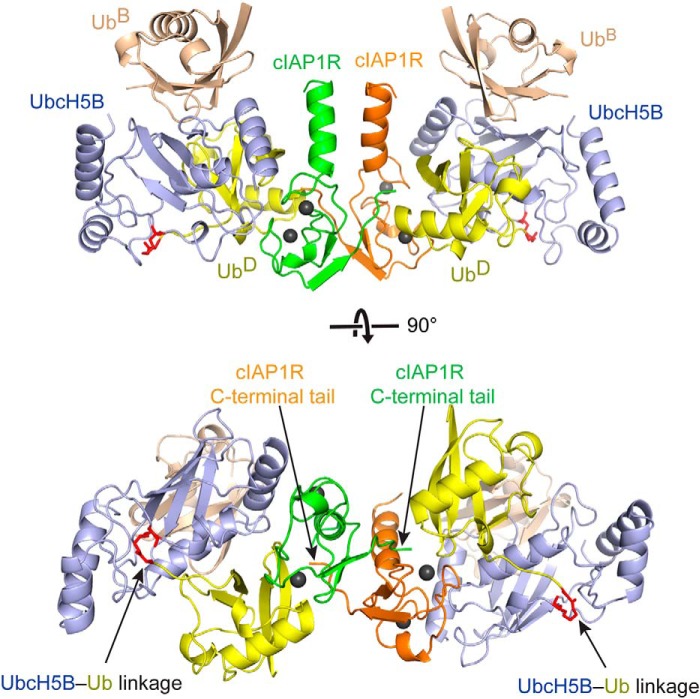
**Crystal structure of cIAP1R-UbcH5B–Ub-Ub^B^ complex.** Shown is a *cartoon representation* of homodimeric cIAP1R-UbcH5B–Ub-Ub^B^ complex generated from crystallographic symmetry. The *top* and *bottom panels* are related by 90° rotation about the *x* axis. The two protomers of cIAP1R are *colored green* and *orange*. UbcH5B is shown in *light blue*, Ub^D^ in *yellow*, and Ub^B^ in *wheat*. Zn^2+^ ions are shown as *gray spheres*. UbcH5B–Ub linkage is shown in *red* and is indicated by *arrows*. cIAP1R's C-terminal tails are indicated by *arrows*.

To gain insight into how Ub^B^ enhances cIAP1R-mediated UbcH5B∼Ub transfer, we crystallized and determined the structure of cIAP1R bound to UbcH5B–Ub and Ub^B^. The cIAP1R-UbcH5B–Ub-Ub^B^ complex crystals belong to space group *C*21 with one copy of cIAP1R-UbcH5B–Ub-Ub^B^ complex in the asymmetric unit. The structure was refined to a resolution of 1.7 Å (Table 2). Because cIAP1 exists as a biological homodimer via the RING domain (6, 18, 40), we used crystallographic symmetry to generate the structure of dimeric cIAP1R-UbcH5B–Ub-Ub^B^ complex (Fig. 2). The structure shows that cIAP1R dimerizes via the RING domain, the C-terminal tail, and a helix that precedes the RING domain similar to other IAP family RING E3s, such as cIAP2, XIAP, and BIRC7 (17, 23, 41). cIAP1R's RING domain binds both UbcH5B and Ub^D^ and stabilizes the UbcH5B–Ub complex in a closed conformation. Additionally, the C-terminal tail of the second subunit in the cIAP1R dimer packs against Ub^D^ in *trans* to stabilize the closed UbcH5B–Ub conformation. These features are similar to those observed in other structures of dimeric RING E3-E2–Ub complexes, such as BIRC7, RNF4, and MDM2-MDMX (22, 23, 30). In our structure, Ub^B^ binds to the backside of UbcH5B centering on the Ser^22^ surface, as reported previously (25, 36).

**Table 2:** **Data collection and refinement statistics**

**Data collection**	**cIAP1R-UbcH5B–Ub-Ub^B^ complex**
Space group	C 1 2 1
Cell dimensions	
*a*, *b*, *c* (Å)	79.19, 53.60, 78.54
α, β, γ (degrees)	90, 107.57, 90
Resolution (Å)	23.52–1.70 (1.74–1.70)
*R*_merge_	0.063 (0.539)*^a^*
*I*/σ	13.8 (2.0)
Completeness (%)	98.8 (94.7)
Redundancy	3.3 (2.7)

**Refinement**	
Resolution (Å)	23.52–1.70
No. of reflections	34,206
*R*_work_/*R*_free_	0.170/0.197
No. of atoms	
Protein	2794
Ions	2
Water	222
*B* factor	
Protein	26.2
Ion	18.2
Water	33.0
RMSDs	
Bond length (Å)	0.007
Bond angles (degrees)	0.922
Ramachandran	
Mostly favored (%)	97.8
Outliers (%)	0

*^a^* Values in parenthesis are for the highest-resolution shell.

### Interactions important for the closed UbcH5B–Ub conformation

Because this is the first structure of cIAP1R bound to E2–Ub, we investigated how cIAP1R stabilizes the closed UbcH5B–Ub conformation to promote Ub transfer. The closed UbcH5B–Ub conformation is stabilized by multiple contacts involving 1) cIAP1R-UbcH5B, 2) cIAP1R-Ub^D^, 3) cIAP1R tail-Ub^D^, and 4) Ub^D^-UbcH5B interactions.

**Figure 3.**
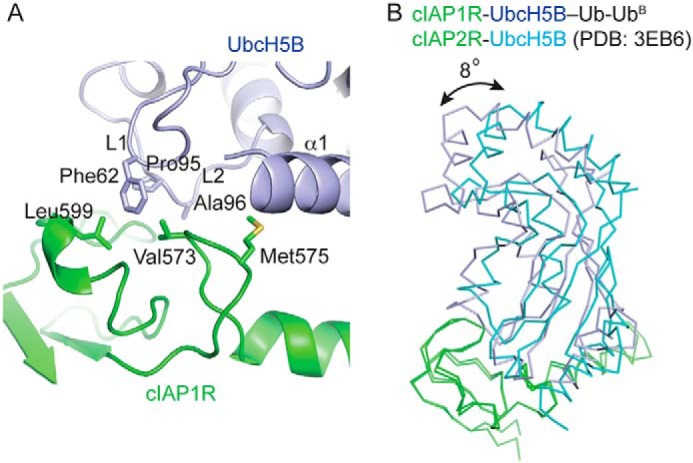
**cIAP1R-UbcH5B interactions.** *A*, *close-up view* of cIAP1R-UbcH5B interactions. UbcH5B's α1, L1, and L2 loops are indicated. All *coloring* is the same as in Fig. 2. *B*, superimposition of cIAP1R portion of structure in cIAP1R-UbcH5B–Ub-Ub^B^ complex with cIAP2R portion of structure in cIAP2R-UbcH5B complex (PDB entry 3EB6). cIAP1R and cIAP2R are *colored* in *green*. UbcH5B from cIAP1R-UbcH5B–Ub-Ub^B^ and cIAP2R-UbcH5B complexes are *colored* in *light blue* and *cyan*, respectively.

The cIAP1R-UbcH5B interaction closely resembled that observed in the structure of cIAP2R-UbcH5B complex (17), which was expected because cIAP1R and cIAP2R share ∼90% sequence identity. The interaction primarily involves cIAP1R's Met^575^ and the hydrophobic core surrounding Val^573^ contacting UbcH5B's α1-helix and L1 and L2 loops (Fig. 3*A*). Despite having nearly identical RING domain sequences, the cIAP1R-UbcH5B portion of the structure and the cIAP2R-UbcH5B structure only superpose with a root mean square deviation (RMSD) of ∼1.0 Å for all Cα atoms. When superimposition was performed using only the RING domain (RMSD of 0.62 Å for Cα atoms), the oblong shape of UbcH5B tilts ∼8°, suggesting subtle differences in UbcH5B-RING domain contacts (Fig. 3*B*). Similar E2 shifts were also observed in the structures of TRAF6 (from human)-Ubc13 and TRAF6 (from *Danio rerio*)-Ubc13–Ub complexes (31, 42). It is unclear whether this E2 movement results from formation of the closed E2–Ub conformation or is due to crystal packing. Nonetheless, the primary RING-E2 interaction is maintained.

**Figure 4.**
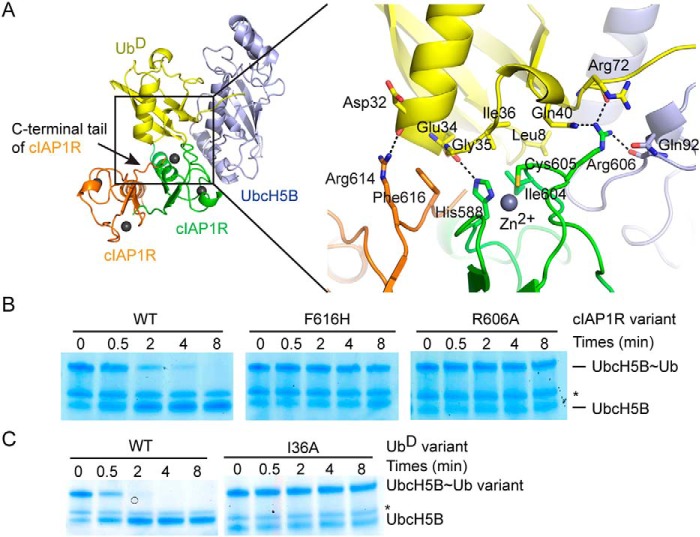
**cIAP1R-Ub^D^ interactions.** *A*, *cartoon representation* of the catalytic competent cIAP1R dimer bound to UbcH5B–Ub (*left*) and *close-up view* of cIAP1R-Ub^D^ interactions (*right*). All *coloring* is the same as in Fig. 2. Hydrogen bonds are shown as *dotted lines. B*, nonreduced SDS-PAGE of lysine discharge reactions showing the disappearance of UbcH5B∼Ub band over time catalyzed by cIAP1R variants. *C*, nonreduced SDS-PAGE of lysine discharge reactions showing the disappearance of UbcH5B∼Ub variant bands over time catalyzed by cIAP1R. *, contaminating band from other reaction components.

Our structure shows that cIAP1R's C-terminal tail, RING domain, and UbcH5B stabilize the closed Ub^D^ conformation. cIAP1R's C-terminal tail interactions involve Arg^614^ and Phe^616^ from the other cIAP1R protomer in the dimer. Arg^614^ forms a hydrogen bond with the carbonyl oxygen of Ub^D^'s Asp^32^, and Phe^616^ packs against Ub^D^'s Gly^35^ surface (Fig. 4*A*). This *trans* tail packing arrangement is similar to those observed in the structures of BIRC7, RNF4, and MDM2-MDMX bound to UbcH5–Ub (22, 23, 30). These RING E3s all contain a Phe or Tyr corresponding to Phe^616^ on cIAP1R that disrupted ligase activity when substituted with histidine or alanine. Likewise, substitution on the corresponding Phe in cIAP2 also disrupted activity (17, 23). To determine the importance of this residue, we mutated cIAP1R's Phe^616^ to His and performed lysine discharge assays to assess the effect on Ub transfer. cIAP1R F616H was defective in discharging UbcH5B∼Ub (Fig. 4*B*), consistent with an earlier study showing that deletion of cIAP1's C-terminal residues abrogates activity (20). Thus, the *trans* tail-Ub^D^ interaction explains the importance of RING domain dimerization.

The cIAP1R-Ub^D^ interactions primarily involve His^588^, Ile^604^, and Cys^605^ from cIAP1R's RING domain contacting Leu^8^ and Ile^36^ patches of Ub^D^. Crucially, cIAP1R's Arg^606^ forms hydrogen bonds with the carbonyl oxygen of Arg^72^ and the side chain of Gln^40^ from Ub^D^ and the carbonyl oxygen of Gln^92^ from UbcH5B (Fig. 4*A*). This Arg^606^ is commonly known as the “linchpin Arg” (33), and its interaction network is conserved in several structures of RING E3-E2–Ub complexes (22–30). To assess the importance of this interaction in cIAP1R, we generated Ub I36A and cIAP1R R606A and tested their effects in UbcH5B∼Ub discharge assays. Although charging of UbcH5B∼Ub I36A was incomplete, as observed previously (23, 25), in the presence of cIAP1R, UbcH5B∼Ub I36A discharged slower than the WT UbcH5B∼Ub (Fig. 4*C*). Similarly, cIAP1R R606A was defective in discharging UbcH5B∼Ub (Fig. 4*B*).

**Figure 5.**
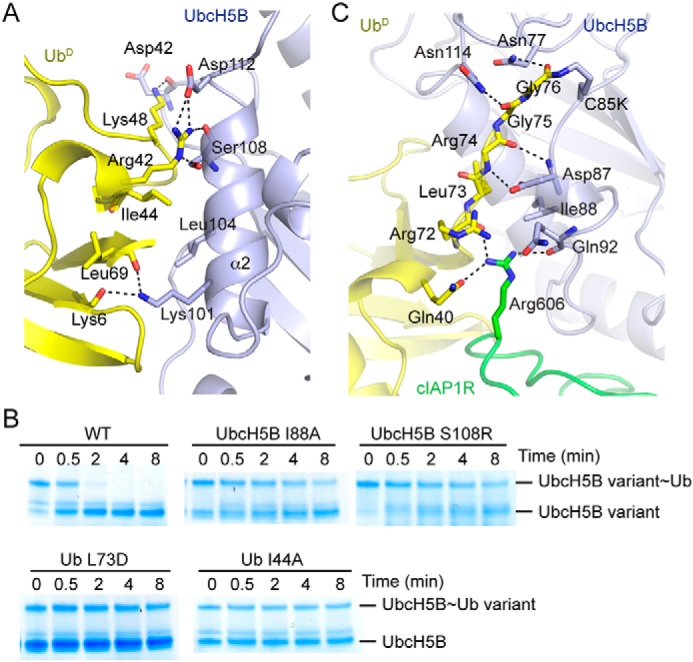
**Ub^D^-UbcH5B interactions.** *A*, *close-up view* of Ub^D^-UbcH5B interactions. *B*, nonreduced SDS-PAGE of lysine discharge reactions showing the disappearance of UbcH5B variant∼Ub or UbcH5B∼Ub variant band over time catalyzed by cIAP1R. *C*, *close-up view* of Ub^D^'s C-terminal tail interactions. All *coloring* in *A* and *B* is the same as in Fig. 2. Hydrogen bonds are shown as *dotted lines* in *A* and *C*.

The Ub^D^-UbcH5B interaction involves Ub^D^'s Ile^44^ patch contacting the Ser^108^ region in UbcH5B's α2-helix (Fig. 5*A*). Additional interactions are also observed between Lys^48^ and Arg^42^ of Ub^D^ and UbcH5B's Asp^42^, Lys^101^, Leu^104^, and Asp^112^ (Fig. 5*A*). To investigate the importance of these interactions, we performed UbcH5B∼Ub discharge assays using Ub I44A and UbcH5B S108R. In both cases, cIAP1R-mediated Ub transfer was impaired (Fig. 5*B*).

The C-terminal tail of Ub^D^ is extended and lies along UbcH5B's active site cleft (Fig. 5*C*). The C-terminal tail of Ub^D^ is stabilized by hydrophobic interactions between UbcH5B's Ile^88^ and Ub^D^'s Leu^73^ and numerous hydrogen bonds involving UbcH5B's Asn^77^, Asp^87^, and Asn^114^ and Ub^D^'s C-terminal tail. To validate the importance of these interactions, we generated Ub L73D and UbcH5B I88A and assessed their effects in UbcH5B∼Ub discharge assays. UbcH5B loaded with Ub L73D and UbcH5B I88A charged with WT Ub were defective in discharge catalyzed by cIAP1R (Fig. 5*B*). Collectively, our data showed that cIAP1R initiates multiple contacts to stabilize UbcH5B–Ub in the closed conformation to promote Ub transfer similar to other RING E3s (22–32).

### Ub^B^-stimulatory mechanism in dimeric cIAP1R-mediated Ub transfer

**Figure 6.**
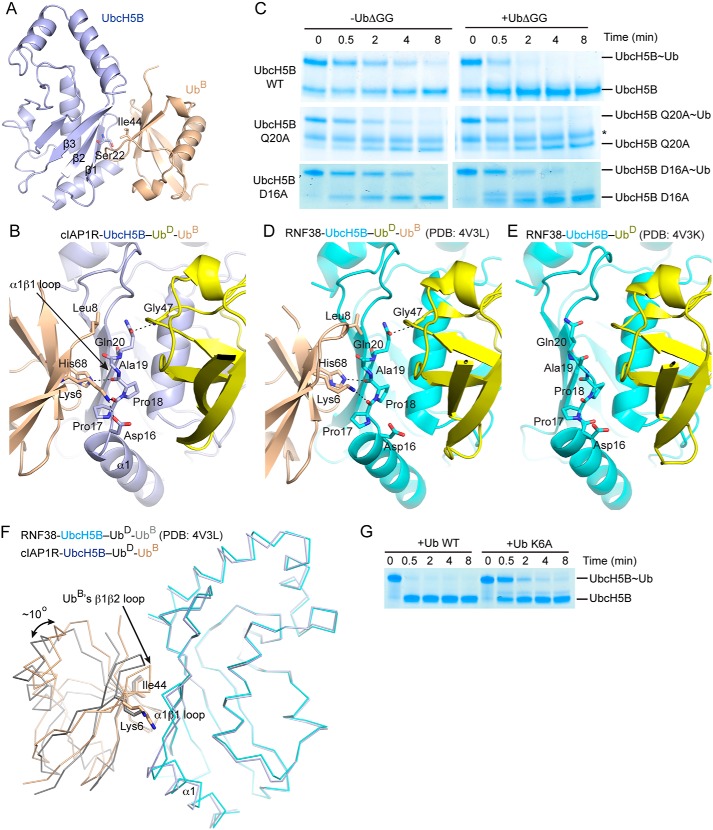
**Ub^B^ interactions.** *A*, *cartoon representation* showing the UbcH5B–Ub^B^ portion of the structure from the cIAP1R-UbcH5B–Ub-Ub^B^ complex. Ile^44^ of Ub^B^ and Ser^22^ of UbcH5B are indicated. *B*, *close-up view* of Ub^B^-UbcH5B–Ub^D^ binding interface. UbcH5B's α1β1-loop is indicated by an *arrow*. Hydrogen bonds are shown as *dotted lines*. All *coloring* in *A* and *B* is the same as in Fig. 2. *C*, nonreduced SDS-PAGE of lysine discharge reactions showing the disappearance of UbcH5B variant∼Ub bands over time in the presence and absence of UbΔGG catalyzed by cIAP1R. *, contaminating band from other reaction components. *D*, close-up view of Ub^B^-UbcH5B–Ub^D^ binding interface in the structure of RNF38-UbcH5B–Ub-Ub^B^ complex (PDB entry 4V3L). UbcH5B is shown in *cyan*, Ub^D^ in *yellow*, and Ub^B^ in *wheat. E*, *close-up view* of UbcH5B's α1β1-loop in the structure of RNF38-UbcH5B–Ub complex (PDB entry 4V3K). UbcH5B is shown in *cyan* and Ub^D^ in *yellow. D* and *E* are shown in the same orientation as in *B. F*, comparison of Ub^B^ conformations in the structures of cIAP1R-UbcH5B–Ub-Ub^B^ and RNF38-UbcH5B–Ub-Ub^B^ complexes (PDB entry 4V3L). Superimposition was performed on all Cα atoms of the UbcH5B portion of the structure. *Ribbon representations* of the UbcH5B-Ub^B^ portion from both structures are shown. Ub^B^'s β1β2 loop is indicated by an *arrow*. UbcH5B and Ub^B^ from cIAP1R-UbcH5B–Ub-Ub^B^ structure are *colored* as in Fig. 2. UbcH5B and Ub^B^ from RNF38-UbcH5B–Ub-Ub^B^ structure are *colored* in *cyan* and *gray*, respectively. *G*, nonreduced SDS-PAGE of lysine discharge reactions showing the disappearance of the UbcH5B∼Ub band over time in the presence of excess WT Ub or Ub K6A catalyzed by cIAP1R.

Ub^B^ binds UbcH5B via the Ile^44^ hydrophobic patch of Ub^B^ and UbcH5B's β1–3 surface surrounding Ser^22^ (Fig. 6*A*). This binding mode resembles other available structures of UbcH5 family E2s bound to Ub^B^ (25, 36, 39, 43). In our structure, Ub^B^ does not contact cIAP1R or Ub^D^ (Fig. 2). In addition to UbcH5B's Ser^22^ surface, Ub^B^ also contacts UbcH5B's α1β1-loop, which in turn packs against Ub^D^ (Fig. 6, *A* and *B*). Here, Ub^B^'s Lys^6^ and His^68^ form hydrogen bonds with carbonyl oxygens of UbcH5B's Pro^17^ and Pro^18^, respectively, and Leu^8^ packs against UbcH5B's Gln^20^, thereby placing Gln^20^ within hydrogen-bonding distance of the backbone amide of Ub^D^'s Gly^47^ (Fig. 6*B*). To test the importance of Gln^20^, we used UbcH5B Q20A to perform cIAP1R-mediated UbcH5B∼Ub discharge assays. The discharge of UbcH5B Q20A∼Ub in the presence and absence of excess of UbΔGG remained similar, suggesting that Gln^20^ plays an important role in Ub^B^-mediated stimulation of Ub transfer (Fig. 6*C*).

Previously, we have determined the structures of a monomeric RING E3, RNF38, bound to UbcH5B–Ub alone and in complex with Ub^B^ (25). These structures showed that in the absence of Ub^B^, UbcH5B's α1β1-loop adopts various conformations that are not optimal for interaction with Ub^D^. The presence of Ub^B^ locks UbcH5B's α1β1-loop into a conformation that helps optimize Ub^D^ for transfer (Fig. 6, *D* and *E*) (25). Superimposition of the structures of cIAP1R-UbcH5B–Ub-Ub^B^ and RNF38-UbcH5B–Ub-Ub^B^ complexes by overlaying the UbcH5B structure reveals that Ub^B^ in cIAP1R-UbcH5B–Ub-Ub^B^ rotates by ∼10° and shifts by ∼1.5–4 Å in different regions across Ub^B^ (Fig. 6*F*). Whereas the Ub^B^ Ile^44^ and UbcH5B Ser^22^ interacting interface is largely maintained, Ub^B^'s β1β2-loop packs more closely to UbcH5B's α1β1-loop in cIAP1R-UbcH5B–Ub-Ub^B^ (Fig. 6*F*). In this manner, Ub^B^'s Lys^6^ moves closer to UbcH5B's α1β1-loop and forms an additional hydrogen bond with UbcH5B's Asp^16^ located at the C terminus of α1; this interaction was not observed in RNF38-UbcH5B–Ub-Ub^B^ (Fig. 6, *B* and *D*). To test the importance of the Ub^B^ Lys^6^-UbcH5B Asp^16^ interaction in Ub^B^-mediated stimulation of Ub transfer, we generated UbcH5B D16A and Ub K6A and performed cIAP1R-mediated UbcH5B∼Ub discharge assays. The discharge of UbcH5B D16A∼Ub remained similar in the presence or absence of excess of UbΔGG (Fig. 6*C*), suggesting that UbcH5B's Asp^16^ plays a role in Ub^B^-mediated stimulation of Ub transfer. Correspondingly, the addition of excess Ub K6A to precharged UbcH5B∼Ub was slower than WT Ub in stimulating cIAP1R-mediated UbcH5B∼Ub discharge (Fig. 6*G*). Thus, the additional contact between Ub^B^ Lys^6^ and UbcH5B Asp^16^ contributes to Ub^B^-mediated stimulation of Ub transfer. Despite this slight difference, the conformation of UbcH5B's α1β1-loop is nearly identical in both structures, which further supports our proposed Ub^B^-stimulatory mechanism, whereby Ub^B^ binding reorganizes UbcH5B's α1β1-loop to help stabilize Ub^D^ in a conformation primed for transfer.

## Discussion

The structure of cIAP1R-UbcH5B–Ub-Ub^B^ reported here provides insight into the Ub^B^-stimulatory mechanism of dimeric RING E3-catalyzed Ub transfer. The cIAP1 RING domain forms a homodimer and utilizes a general mechanism that is shared by other RING E3s to stabilize UbcH5B–Ub in a closed conformation to activate the thioester bond for catalysis (3). Ub^B^ functions by reorganizing UbcH5B's α1β1-loop conformation to reinforce Ub^D^ in the closed conformation, thereby enhancing Ub transfer in a manner consistent with our prior study with the monomeric RING E3 RNF38 (25). Our current work demonstrates that the Ub^B^-stimulatory mechanism is conserved in both monomeric and dimeric RING E3-catalyzed reactions with the UbcH5 family of E2s.

The closed E2∼Ub conformation has been shown to be important for Ub transfer, and the role of the RING domain is to promote the transition to this conformation to enhance the rate of Ub transfer (22, 23, 33, 44, 45). In addition to the established contacts between RING-E2, RING-Ub^D^, and Ub^D^-E2, several RING E3s have evolved different mechanisms to facilitate this process (3). For cIAP1, the RING dimer arrangement enables cIAP1R to utilize the C-terminal tail of the other dimeric cIAP1R protomer to stabilize Ub^D^. This mechanism is observed in several dimeric RING E3s containing a Phe or Tyr residue in their C-terminal tail, such as BIRC7, RNF4, and MDM2-MDMX (22, 23, 30).

Noncovalent Ub binding to the backside of UbcH5 family E2 has been shown to increase the processivity of Ub transfer (25, 36–39). Mechanistically, we have recently shown that Ub^B^ binding improved RING E3's affinity for the E2∼Ub complex and that the RING E3-E2∼Ub complex displayed higher affinity for Ub^B^ using the monomeric RING E3 RNF38 (25). Here we observed a similar synergistic effect with the dimeric RING E3, cIAP1. We have shown previously that the *K_d_* for the Ub^B^-UbcH5B interaction was ∼280 μm (25). In the presence of the cIAP1R, UbcH5B–Ub complex is primed into the closed conformation, and the *K_d_* for Ub^B^-UbcH5B binding improved to ∼13 μm (Table 1). Our structure showed that the closed UbcH5B–Ub conformation stabilizes UbcH5B's α1β1-loop, which in turn forms optimal interaction with Ub^B^ and could explain the drop in *K_d_*. The total cellular Ub concentration is ∼20–85 μm, depending on cell type. Within this total concentration, Ub presents as a mixture of monoubiquitinated substrates, free Ub, thioester intermediates of ligation machinery, and poly-Ub chains (46, 47). A previous study (25) and our current study showed that these forms of Ub can serve as sources of Ub^B^, and hence the total cellular Ub concentration could serve as the guide for the availability of Ub^B^. The formation of cIAP1-UbcH5B∼Ub complex lowers the *K_d_* for the Ub^B^-UbcH5B interaction to a value in which the Ub^B^ interaction would be favorable in cells. We anticipate that noncovalent Ub binding would have an impact on cIAP1-UbcH5–catalyzed ubiquitination in cells. In both crystal structures of cIAP1R-UbcH5B–Ub-Ub^B^ and RNF38-UbcH5B–Ub-Ub^B^ (25) complexes, Ub^B^ alters UbcH5B's α1β1-loop into a nearly identical configuration to buttress Ub^D^ in the closed conformation. The subtle differences in Ub^B^ conformations seen in the two structures could potentially arise from crystal packing. Nonetheless, the cIAP1R-UbcH5B–Ub-Ub^B^ structure presented here provides a more detailed view of how Ub^B^ could make an additional contact with UbcH5B's α1 C terminus and α1β1-loop to optimize these elements in stabilizing the closed Ub^D^ conformation. In conclusion, our work shows that Ub^B^ serves as an allosteric activator of RING E3-E2∼Ub complexes and that the Ub^B^-stimulatory mechanism is conserved for both monomeric and dimeric RING E3s.

## Experimental procedures

### Protein expression and purification

All constructs were expressed in *Escherichia coli* BL21 (DE3) Gold (Stratagene). All proteins used are from humans unless otherwise specified. cIAP1 RING domain (residues 556-C; cIAP1R) was cloned into pGEX4T1 (GE Healthcare), which contains an N-terminal GST tag followed by a tobacco etch virus protease cleavage site. cIAP1R was purified by GSH affinity chromatography, followed by tobacco etch virus cleavage to release the GST tag. The released GST tag was removed by GSH affinity chromatography, and the cleaved cIAP1R was purified by size-exclusion chromatography. *Arabidopsis thaliana* Uba1, untagged UbcH5B variants, ^32^P-Ub, Ub, and Ub lacking the C-terminal diglycine motif (UbΔGG) were prepared as described previously (25). Fluorescently labeled Ub was prepared as described previously (30). UbcH5B–Ub, UbcH5B_S22R_–Ub, and UbcH5B S22R,F62A,P95D–Ub were generated and purified as described previously (25). Protein concentrations were determined by Bradford assay using BSA as a standard. Ub concentration was determined by measuring the absorbance at 280 nm and the molar extinction coefficient calculated from the protein sequence. Proteins were stored in 25 mm Tris-HCl (pH 7.6), 0.15 m NaCl, and 1 mm DTT at −80 °C.

### Crystallization

cIAP1R-UbcH5B–Ub-Ub^B^ complex was assembled by mixing cIAP1R (8.5 mg/ml), UbcH5B–Ub (20 mg/ml), and Ub (100 mg/ml) at 1:1:1.2 molar ratio. Crystals were obtained by mixing protein complex with an equal volume of reservoir solution containing 0.2 m ammonium fluoride and 15% (w/v) PEG 3350 using sitting drop vapor diffusion at 19 °C. The crystals were harvested and flash-frozen in 0.2 m ammonium fluoride, 18% (w/v) PEG 3350, and 20% (v/v) ethylene glycol.

### Data collection and processing

Data were collected at beamline I03 at Diamond Light Source, processed using xia2 pipeline (48), and integrated with automated XDS (49). Initial phases of cIAP1R-UbcH5B–Ub-Ub^B^ complex were obtained by molecular replacement with PHASER (50) using UbcH5B and Ub from PDB entry 3ZNI and cIAP2 RING from PDB entry 3EB6. All models were built in COOT (51) and refined using PHENIX (52). cIAP1R-UbcH5B–Ub-Ub^B^ complex was refined to a resolution of 1.7 Å. The final model contains one copy of cIAP1R (chain A, residues 556-C), one copy of Ub^B^ (chain B residues 1–74), one copy of UbcH5B (chain C residues 2–147), and one copy of Ub^D^ (chain D, residues 1–76). All figure models were generated using PyMOL.

### Lysine discharge assays

UbcH5B variant (15 μm) was charged with equimolar Ub variant (15 μm), ^32^P-Ub (15 μm), or fluorescently labeled Ub (15 μm) in a reaction containing 50 mm Tris-HCl, pH 7.6, 50 mm NaCl, *Arabidopsis* Uba1 (1 μm), BSA (1 mg/ml), 5 mm MgCl_2_, and 5 mm ATP for 15 min at 23 °C as described previously (25). The charged reaction was stopped by adding 0.01 units/ml apyrase and 30 mm EDTA for 2 min at 23 °C. The lysine discharge reaction was initiated by adding a mixture containing 50 mm Tris-HCl, pH 7.6, 50 mm NaCl, BSA (1 mg/ml), l-lysine (20 mm), and cIAP1R variant (0.5 μm) in the presence or absence of UbΔGG (300 μm for Figs. 1*A*, 4 (*B* and *C*), 5*B*, and 6*C*; 20 μm for Fig. 1*B*) or UbcH5B S22R,F62A,P95D–Ub (20 μm; Fig. 1*B*). WT Ub (300 μm) and K6A Ub (300 μm) were used to perform lysine discharge assays in Fig. 6*G*. Final concentrations are shown in parenthesis except for UbcH5B and Ub variants, which were ∼5 μm. Reactions were quenched with 2× SDS-loading buffer at the indicated time points and resolved by SDS-PAGE and visualized by staining with InstantBlue. Reactions performed using ^32^P-Ub were dried and visualized using autoradiography. Fluorescently labeled UbcH5B∼Ub was visualized by a LI-COR Odyssey scanner, followed by staining with InstantBlue.

### SPR

All SPR experiments were performed at 25 °C on a Biacore T200 system with a CM-5 chip. For cIAP1R-UbcH5B and cIAP1R-UbcH5B–Ub variant binding experiments, GST-cIAP1R was coupled to CM-5 chips as described previously (25). UbcH5B and UbcH5B–Ub variants were serially diluted in running buffer containing 25 mm Tris-HCl, pH 7.6, 150 mm NaCl, 0.1 mg/ml BSA, 1 mm DTT, and 0.005% (v/v) Tween 20. For experiments with UbΔGG, UbcH5B–Ub variants were serially diluted in running buffer containing 0.6 mm UbΔGG. For the Ub^B^-UbcH5B backside binding experiment, GST-Ub was captured on a CM-5 chip, and UbcH5B–Ub was mixed with a 2.4-fold molar excess of cIAP1R (100 μm UbcH5B–Ub and 240 μm cIAP1R) and then serially diluted in running buffer containing 10 μm cIAP1R to ensure that all UbcH5B–Ub concentration ranges were saturated with cIAP1R (cIAP1R binds UbcH5B–Ub with a *K_d_* of ∼0.83 μm; Table 1). Binding was measured at the indicated concentration ranges as in Fig. S1. Data reported are the differences in SPR signal between GST-cIAP1R and GST alone or GST-Ub and GST alone. The data were analyzed by steady-state affinity analysis using Biacore T200 BIAevaluation software (GE Healthcare) and Scrubber2 (BioLogic Software).

## Author contributions

A. P. purified proteins and performed ubiquitination assays and crystallization. G. S. performed and analyzed SPR experiments. A. P. and D. T. H. determined the structure and wrote the manuscript.

## Supplementary Material

Supporting Information

## Footnotes

2 The abbreviations used are:

Ub: ubiquitin
PDB: Protein Data Bank
cIAP1 and cIAP2: cellular inhibitor of apoptosis protein-1 and -2, respectively
IAP: inhibitor of apoptosis
E1: Ub-activating enzyme
E2: Ub-conjugating enzyme
E3: Ub-ligase
E2∼Ub: E2∼ubiquitin conjugate
UbcH5B–Ub: UbcH5B covalently linked to Ub
RING: really interesting new gene
HECT: homologous to E6-AP C terminus
SMAC: second mitochondria-derived activator of caspase
XIAP: X-linked inhibitor of apoptosis protein
TRAF: TNF receptor–associated factor protein
RIPK1: receptor-interacting serine/threonine-protein kinase 1
UBA: Ub-associated domain
BIR: baculoviral IAP repeat domain
CARD: caspase-recruiting domain
Ub^B^: backside-bound Ub
Ub^D^: donor Ub
cIAP1R: cIAP1 RING domain
SPR: surface plasmon resonance
RSMD: root mean square deviation
RBR: RING-in-between-RING
GST: glutathione *S*-transferase.
