# Supplementary material for: Structural insights into non-covalent ubiquitin activation of the cIAP1-UbcH5B∼ubiquitin complex
Source: J Biol Chem. 2018 Dec 6;294(4):1240–9. doi: 10.1074/jbc.RA118.006045 (PMC6349121; doi:10.1074/jbc.RA118.006045)
Supplement: Supporting Information [file supp_294_4_1240__index.html]

Structural insights into non-covalent ubiquitin activation of the cIAP1-UbcH5B~ubiquitin complex — Activation of UbcH5B~Ub by cIAP1 and non-covalent ubiquitin — Structural insights into non-covalent ubiquitin activation of the cIAP1-UbcH5B∼ubiquitin complex — Activation of UbcH5B∼Ub by cIAP1 and non-covalent ubiquitin — Supporting Information 

# Structural insights into non-covalent ubiquitin activation of the cIAP1-UbcH5B∼ubiquitin complex

## Supporting Information

- Supporting Information (to be published online) - Figure S1
