# Supplementary material for: Structural insights into non-covalent ubiquitin activation of the cIAP1-UbcH5B∼ubiquitin complex
Source: J Biol Chem. 2018 Dec 6;294(4):1240–9. doi: 10.1074/jbc.RA118.006045 (PMC6349121; doi:10.1074/jbc.RA118.006045)
Supplement: Supporting Information [file supp_RA118.006045_141039_2_supp_242698_p7yryk.pdf]

SUPPORTING INFORMATION

Structural insights into non-covalent ubiquitin activation of the cIAP1-UbcH5B~ubiquitin complex

Amrita Patel, Gary J. Sibbet and Danny T. Huang

Cancer Research UK Beatson Institute, Garscube Estate, Switchback Road, Glasgow G61 1BD, United Kingdom and Institute of Cancer Sciences, University of Glasgow, Glasgow, G61 1BD, United Kingdom.

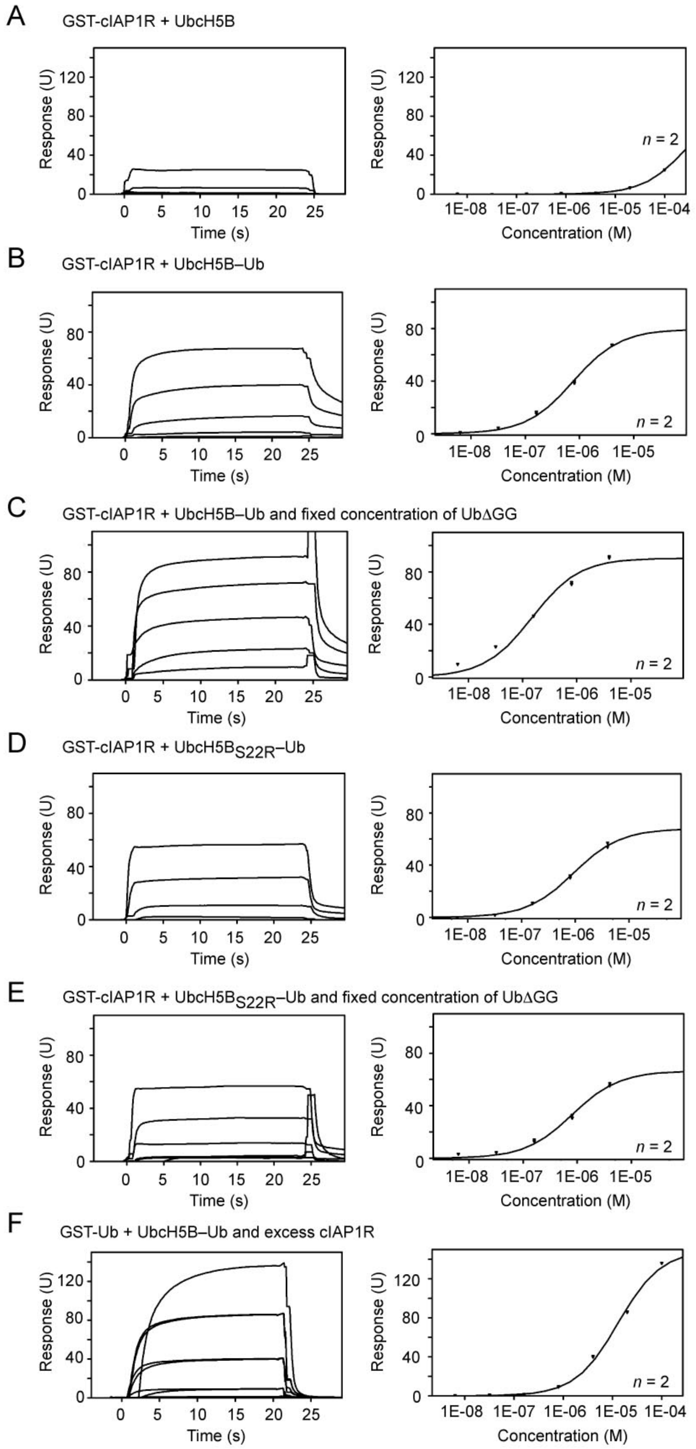

Figure S1. SPR analyses of GST-cIAP1R and GST-Ub binding affinities for UbcH5B and UbcH5B–Ub variants alone and in the presence of UbΔGG or cIAP1R, related to Table 1. Representative sensorgrams (left) and binding curves (right) for (**A**) GST-cIAP1R and UbcH5B, (B) GST-cIAP1R and UbcH5B–Ub, (C) GST-cIAP1R and UbcH5B in the presence of 0.6 mM UbΔGG, (D) GST-cIAP1R and UbcH5B$_{S22R}$–Ub, (E) GST-cIAP1R and UbcH5B$_{S22R}$–Ub in the presence of 0.6 mM UbΔGG and (F) GST-Ub and UbcH5B–Ub in the presence of excess cIAP1R. Number of replicates is indicated in the binding curve.
